# Supplementary material for: Diagnostic accuracy of the lumbar spinal stenosis-diagnosis support tool and the lumbar spinal stenosis-self-administered, self-reported history questionnaire
Source: PLoS One. 2022 May 5;17(5):e0267892. doi: 10.1371/journal.pone.0267892 (PMC9070893; doi:10.1371/journal.pone.0267892)
Supplement: S1 Table — In this analysis, participants with >7 points on the LSS-DST, despite missing ABI and other values, were treated as LSS-DST-positive, and participants with <7 points, despite missing ABI and other values, were treated as LSS-DST-negative (n = 7,914). ABI, ankle brachial index; CI, confidence interval; DST, diagnosis support tool; LSS, lumbar spinal stenosis; NASS, North American Spine Society; SSHQ, self-administered, self-reported history questionnaire. (DOCX) [file pone.0267892.s002.docx]

**S2 Table.** Sensitivity and specificity of the NASS clinical description of LSS, LSS-DST, and LSS-SSHQ

| Index test | Sensitivity | | *P*-value for heterogeneity | Specificity | | *P*-value for heterogeneity |
| --- | --- | --- | --- | --- | --- | --- |
|  | Point estimate | (95% CI) |  | Point estimate | (95% CI) |  |
| 1) NASS clinical description of LSS | 59.5% | 57.8%–61.3% |  | 91.2% | 90.4%–92.0% |  |
| 2) LSS-DST | 90.2% | 89.1%–91.2% | 2) vs. 1) <0.0001 | 82.9% | 81.9%–84.0% | 2) vs. 1) <0.0001 |
| 3) LSS-SSHQ | 84.4% | 83.1%–85.7% | 3) vs. 1) <0.0001 | 61.7% | 60.3%–63.0% | 3) vs. 1) <0.0001 |

In this analysis, participants with >7 points on the LSS-DST, despite missing ABI and other values, were treated as LSS-DST-positive, and participants with <7 points, despite missing ABI and other values, were treated as LSS-DST-negative (n = 7,914).

ABI, ankle brachial index; CI, confidence interval; DST, diagnosis support tool; LSS, lumbar spinal stenosis; NASS, North American Spine Society; SSHQ, self-administered, self-reported history questionnaire
