# Supplementary material for: Diagnostic accuracy of the lumbar spinal stenosis-diagnosis support tool and the lumbar spinal stenosis-self-administered, self-reported history questionnaire
Source: PLoS One. 2022 May 5;17(5):e0267892. doi: 10.1371/journal.pone.0267892 (PMC9070893; doi:10.1371/journal.pone.0267892)
Supplement: S2 Table — In this analysis, participants with >7 points on the LSS-DST, despite missing ABI and other values, were treated as LSS-DST-positive, and participants with <7 points, despite missing ABI and other values, were treated as LSS-DST-negative (n = 7,914). ABI, ankle brachial index; CI, confidence interval; DORs, diagnostic odds ratios; DST, diagnosis support tool; LSS, lumbar spinal stenosis; NASS, North American Spine Society; SSHQ, self-administered, self-reported history questionnaire. (DOCX) [file pone.0267892.s003.docx]

**S3 Tabl**e. DORs of the NASS clinical description of LSS, LSS-DST, and LSS-SSHQ

| Index test | DOR | |
| --- | --- | --- |
|  | Point estimate | (95% CI) |
| 1) NASS clinical description of LSS | 15.3 | 13.5–17.3 |
| 2) LSS-DST | 44.5 | 38.6–51.3 |
| 3) LSS-SSHQ | 8.7 | 7.8–9.8 |

In this analysis, participants with >7 points on the LSS-DST, despite missing ABI and other values, were treated as LSS-DST-positive, and participants with <7 points, despite missing ABI and other values, were treated as LSS-DST-negative (n = 7,914).

ABI, ankle brachial index; CI, confidence interval; DORs, diagnostic odds ratios; DST, diagnosis support tool; LSS, lumbar spinal stenosis; NASS, North American Spine Society; SSHQ, self-administered, self-reported history questionnaire
