# Supplementary material for: Diagnostic accuracy of the lumbar spinal stenosis-diagnosis support tool and the lumbar spinal stenosis-self-administered, self-reported history questionnaire
Source: PLoS One. 2022 May 5;17(5):e0267892. doi: 10.1371/journal.pone.0267892 (PMC9070893; doi:10.1371/journal.pone.0267892)
Supplement: S3 Table — CI, confidence interval; DST, diagnosis support tool; LSS, lumbar spinal stenosis; NASS, North American Spine Society; SSHQ, self-administered, self-reported history questionnaire. (DOCX) [file pone.0267892.s004.docx]

**S4 Table.** Sensitivity and specificity of the NASS clinical description of LSS, LSS-DST, and LSS-SSHQ in participants aged >60 years (n=2,136)

| Index test | Sensitivity | | *P*-value for heterogeneity | Specificity | | *P*-value for heterogeneity |
| --- | --- | --- | --- | --- | --- | --- |
|  | Point estimate | (95% CI) |  | Point estimate | (95% CI) |  |
| 1) NASS clinical description of LSS | 64.3% | 61.7%–67.0% |  | 83.3% | 80.9%–85.8% |  |
| 2) LSS-DST | 91.7% | 90.2%–93.2% | 2) vs. 1) <0.0001 | 57.8% | 54.6%–61.1% | 2) vs. 1) <0.0001 |
| 3) LSS-SSHQ | 83.6% | 81.5%–85.6% | 3) vs. 1) <0.0001 | 49.9% | 46.6%–53.2% | 3) vs. 1) <0.0001 |

CI, confidence interval; DST, diagnosis support tool; LSS, lumbar spinal stenosis; NASS, North American Spine Society; SSHQ, self-administered, self-reported history questionnaire
