# Supplementary material for: Diagnostic accuracy of the lumbar spinal stenosis-diagnosis support tool and the lumbar spinal stenosis-self-administered, self-reported history questionnaire
Source: PLoS One. 2022 May 5;17(5):e0267892. doi: 10.1371/journal.pone.0267892 (PMC9070893; doi:10.1371/journal.pone.0267892)
Supplement: S4 Table — CI, confidence interval; DORs, diagnostic odds ratios; DST, diagnosis support tool; LSS, lumbar spinal stenosis; NASS, North American Spine Society; SSHQ, self-administered, self-reported history questionnaire. (DOCX) [file pone.0267892.s005.docx]

**S5 Table.** DORs of the NASS clinical description of LSS, LSS-DST, and LSS-SSHQ in participants aged >60 years (n=2,136)

| Index test | DOR | |
| --- | --- | --- |
|  | Point estimate | (95% CI) |
| 1) NASS clinical description of LSS | 9.0 | 7.3–11.1 |
| 2) LSS-DST | 15.2 | 11.9–19.3 |
| 3) LSS-SSHQ | 5.1 | 4.2–6.2 |

CI, confidence interval; DORs, diagnostic odds ratios; DST, diagnosis support tool; LSS, lumbar spinal stenosis; NASS, North American Spine Society; SSHQ, self-administered, self-reported history questionnaire
