# Supplementary material for: Diagnostic accuracy of the lumbar spinal stenosis-diagnosis support tool and the lumbar spinal stenosis-self-administered, self-reported history questionnaire
Source: PLoS One. 2022 May 5;17(5):e0267892. doi: 10.1371/journal.pone.0267892 (PMC9070893; doi:10.1371/journal.pone.0267892)
Supplement: S5 Table — Each comparison was subjected to a χ-square test. LSS, lumbar spinal stenosis. (DOCX) [file pone.0267892.s006.docx]

**S6 Table.** Comparison of the participants who were included and those who were excluded

| Characteristic | Participants who were included | Participants who were Excluded | *P*-value |
| --- | --- | --- | --- |
|  | n(%) | n(%) |  |
| Age (years) |  |  | 0.0008 |
| 20-29 | 166(5.0) | 420(5.7) |  |
| 30-39 | 310(9.3) | 780(10.6) |  |
| 40-40 | 308(9.3) | 792(10.8) |  |
| 50-59 | 411(12.3) | 976(13.3) |  |
| 60-69 | 892(26.8) | 1824(24.9) |  |
| 70+ | 1244(37.3) | 2546(34.7) |  |
| Sex |  |  | <0.0001 |
| Male | 1755(52.9) | 3576(48.8) |  |
| Female | 1564(47.1) | 3756(51.2) |  |
| Missing data | 12 | 6 |  |
| Presence of LSS |  |  | <0.0001 |
| LSS(-) | 1915(57.5) | 5223(71.2) |  |
| LSS(+) | 1416(42.5) | 2155(28.8) |  |

Each comparison was subjected to a χ-square test.

LSS, lumbar spinal stenosis
